# Remote Patient Monitoring for Neuropsychiatric Disorders: A Scoping Review of Current Trends and Future Perspectives from Recent Publications and Upcoming Clinical Trials

**DOI:** 10.1089/tmj.2021.0489

**Published:** 2022-09-07

**Authors:** Tetsuo Sakamaki, Yoshihiko Furusawa, Ayako Hayashi, Masaru Otsuka, Jovelle Fernandez

**Affiliations:** ^1^ Medical Informatics and Decision Sciences, Gunma University Graduate School of Medicine, Maebashi, Japan.; ^2^ Japan Medical Office, Takeda Pharmaceutical Company Limited, Tokyo, Japan.; ^3^ Enterprise Digital Lead, Takeda Pharmaceutical Company Limited, Tokyo, Japan.

**Keywords:** neuropsychiatry, telemedicine, wearable electronic devices, e-Health, monitoring, telehealth

## Abstract

**Introduction:**

Telemedicine and remote patient monitoring are rapidly growing fields. This scoping review provides an update on remote patient monitoring for neuropsychiatric disorders from recent publications and upcoming clinical trials.

**Methods:**

Publications (PubMed and ICHUSHI; published January 2010 to February 2021) and trials (ClinicalTrials.gov and Japanese registries; active or recruiting by March 2021) that assessed wearable devices for remote management and/or monitoring of patients with neuropsychiatric disorders were searched. The review focuses on disorders with ≥3 publications.

**Results:**

We identified 44 publications and 51 active or recruiting trials, mostly from 2019 or 2020. Research on digital devices was most common for Parkinson's disease (11 publications and 19 trials), primarily for monitoring motor symptoms and/or preventing falls. Other disorders (3–5 publications each) included epilepsy (electroencephalogram [EEG] and seizure prediction), sleep disorder (sleep outcomes and behavioral therapies), multiple sclerosis (physical activity and symptoms), depression (physical activity, symptoms, and behavioral therapies), and amyotrophic lateral sclerosis (symptoms). Very few studies focused on newly emerging technologies (e.g., in-ear EEG and portable oximeters), and few studies integrated remote symptom monitoring with telemedicine.

**Discussion:**

Currently, development of digital devices for daily symptom monitoring is focused on Parkinson's disease. For the diseases reviewed, studies mostly focused on physical activity rather than psychiatric or nonmotor symptoms. Although the validity and usefulness of many devices are established, models for implementing remote patient monitoring in telehealth settings have not been established.

**Conclusions:**

Verification of the clinical effectiveness of digital devices combined with telemedicine is needed to further advance remote patient care for neuropsychiatric disorders.

## Introduction

Monitoring of patient symptoms in home-based settings has become commonplace for long-term monitoring of chronic diseases.^1,2^ Movement trackers and physiological/biochemical sensors (e.g., for blood pressure, heart rate, body temperature, and blood sugar) are routinely applied in home-based settings for many conditions, including heart disease, hypertension, and diabetes.^2^ It is well known that with the recent evolution of wearable digital technologies and virtual communication, it is now possible to integrate remote patient monitoring into health care delivery.^2^ Moreover, travel and safety restrictions during the coronavirus disease 2019 (COVID-19) pandemic have given renewed impetus to the integration of remote patient monitoring with telemedicine.^3,4^

Many studies from around the world have assessed the use of telemedicine for neurological and psychiatric disorders,^5,6^ primarily because of the substantial burden these diseases impart.^7,8^ Patients often experience lifelong disabling symptoms and progressive deterioration in cognition, physical function, and/or mental health, all of which have significant negative effects on the well-being and quality of life of patients and carers. Measurement of disease progression entails both objective measures and subjective clinician- and patient-reported outcomes related to functions, symptoms, and health-related quality of life.^9–12^

For patients with chronic neurological disorders, most research is focused on physical activity as a measure of symptom deterioration and treatment effectiveness and to predict adverse events.^5^ For example, because of the subjective nature of clinical assessment and because patients typically present with a complex array of symptoms, continuous objective monitoring of symptoms and adverse events has the potential to provide real-time information that can help guide the timing of drug administration to improve outcomes and help reduce side effects.^5,6^ Despite these advances, integration of remote patient monitoring with telemedicine remains a challenge now and into the future.

In this review, we conducted a comprehensive literature search of publications and upcoming clinical trials on the use of wearable devices for routine patient monitoring in neuropsychiatric disorders. A scoping approach was taken to identify (1) recent publications from the peer-reviewed literature to gain an understanding of the focus of current research and (2) active or actively recruiting clinical trials to anticipate future trends. The objective of our review was to identify the extent to which monitoring and visualization of neuropsychiatric symptoms are being validated and used in conjunction with telehealth in many countries rather than on the development of devices *per se*. In particular, the review is focused on the most recent trends and opportunities with regard to remote patient monitoring and the identification of studies that integrate remote patient monitoring with delivery of online medical care.

## Methods

### STUDY DESIGN, DATABASES, AND SEARCH TERMS

This was a scoping review of peer-reviewed publications in PubMed and ICHUSHI and the following online clinical trial databases: ClinicalTrials.gov (English), the Japan Pharmaceutical Information Center Clinical Trials Information website, the University Hospital Medical Information Network Center Clinical Trials Registry, the Japan Registry of Clinical Trials, and the Japan Medical Association Center for Clinical Trials website. The search strategies and search terms were tailored for each database.

Search terms included Medical Subject Headings (MeSH) for PubMed, MeSH terms converted to thesaurus terms for ICHUSHI, and free-text terms connected by Boolean operators (AND, OR). The search terms and search hierarchy are described in detail in *Supplementary Tables S1 and S2*. As this was a literature review of publicly available information, institutional review board approval was not required. Although we took a methodical approach to the development of literature search, a formal protocol was not prepared for this scoping review.

### INCLUSION AND EXCLUSION CRITERIA

Publications and clinical trials on neurological or psychiatric disorders were included if they assessed wearable, portable, or mountable devices (1) that are intended for at-home monitoring to facilitate disease management or (2) for remote management of patients. Studies that did not provide information on the clinical use of wearable devices or that were only focused on the development of software algorithms for digital devices were excluded.

PubMed and ICHUSHI searches were limited to full-text publications with abstracts that were published between January 1, 2010, and February 12, 2021, and included any neurological or psychiatric disorder. Review articles were excluded.

Clinical trial searches (ClinicalTrials.gov and Japanese databases) were conducted on March 8, 2021, and were limited to trials that were active or actively recruiting. Searches in each clinical trial database included the neuropsychiatric disorders for which three or more publications had been retrieved from the literature search (i.e., Parkinson's disease, epilepsy, sleep disorder, multiple sclerosis, depression, and amyotrophic lateral sclerosis [ALS]).

### SCREENING AND FINAL ELIGIBILITY

Initial electronic screening of each publication/trial record was conducted to identify potential studies using the following criteria: (1) use of a wearable, home, or portable device for patient monitoring; (2) patients with a neuropsychiatric disorder; and (3) studies focused on the remote use of wearable devices. Inclusion of each study was confirmed after a manual review of the full text or full record; duplicate publications and clinical trials were excluded manually.

Four reviewers conducted the screening. Reviewer 1 sequentially evaluated the titles and abstracts of publications identified by the online literature searches, and then manually assessed the full text of all potential publications against the eligibility criteria. Reviewer 2 screened the full electronic clinical trial records for all trials that were retrieved by the online search against the eligibility criteria. Reviewers 3 and 4 confirmed the eligibility of all publications and clinical trials identified by Reviewers 1 and 2 against eligibility criteria. Any discrepancy was resolved by consensus.

### DATA CHARTING

Data from each eligible publication and clinical trial were charted into prespecified spreadsheets by Reviewers 1 and 2, respectively, and all chartered data were checked by Reviewers 3 and 4 against the original source. Inconsistencies between the initial data extraction and a reviewer were verified from the original source.

For publications, the following data were charted: citation information, title, disease, study location, abstract, objective, study design, patient population, comparators, eligibility criteria, intervention, device name, device overview, time frame, outcomes measured using the digital device, and assessment of telemedicine. For clinical trials, the following data were charted: title, disease, synopsis, objective, study design, target sample size, eligibility criteria, intervention, comparators, primary and secondary outcomes, sponsor, device name, device overview, study location, trial status, trial ID, registration date, start date, primary completion date, and completion date.

### DATA SYNTHESIS

Data charted from each publication and clinical trial were grouped by neuropsychiatric disease. The numbers of publications and clinical trials that met the eligibility criteria were counted and the dates of publication or trial registration were summarized. For each neuropsychiatric disease, objectives and/or intended uses of the digital device (for publications) or study objectives (clinical trials) were tabulated and summarized. Current trends and future perspectives and the role of telemedicine for each neuropsychiatric disease were tabulated and summarized.

## Results

### STUDIES RETRIEVED

**Fig. 1.**
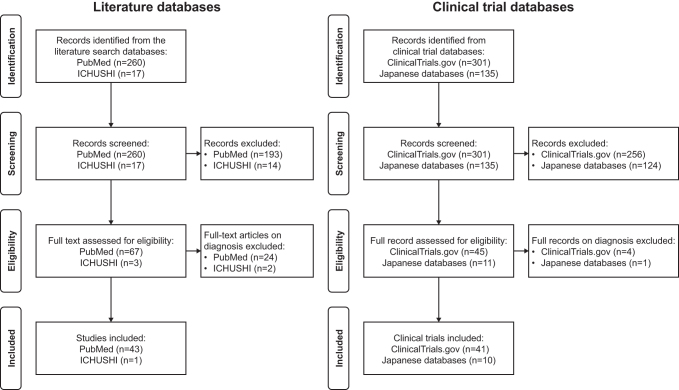
Flowchart summarizing the results of search strategies.

**Fig. 2.**
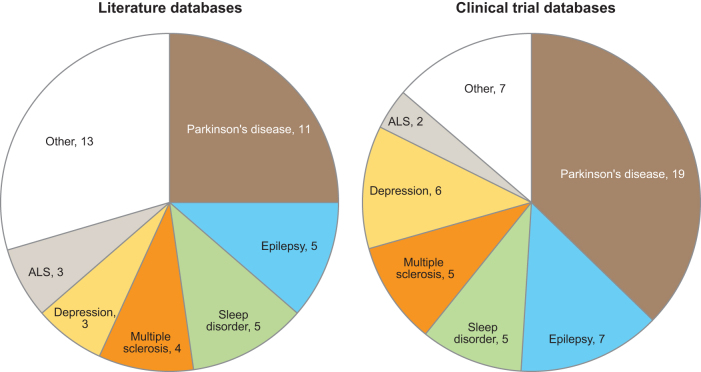
Number of publications (*N* = 44) and active or actively recruiting clinical trials (*N* = 51) included in this review by neuropsychiatric disorder.

A total of 95 studies were retrieved, which included 44 publications (43 from PubMed and 1 from ICHUSHI) and 51 active or actively recruiting clinical trials (41 from ClinicalTrials.gov and 10 from Japanese databases) (*Fig. 1*). Of the 44 publications, the most common neuropsychiatric disorders were Parkinson's disease followed by epilepsy, sleep disorder, multiple sclerosis, depression, and ALS (*Fig. 2*). Other neuropsychiatric disorders included dementia (2 publications), stroke (2 publications), autism spectrum disorders, hyperactivity behavior, intracranial hypertension, hydrocephalus, schizophrenia, essential tremor, Gaucher disease, neurovascular disorders, and fibromyalgia (1 publication each).

**Fig. 3.**
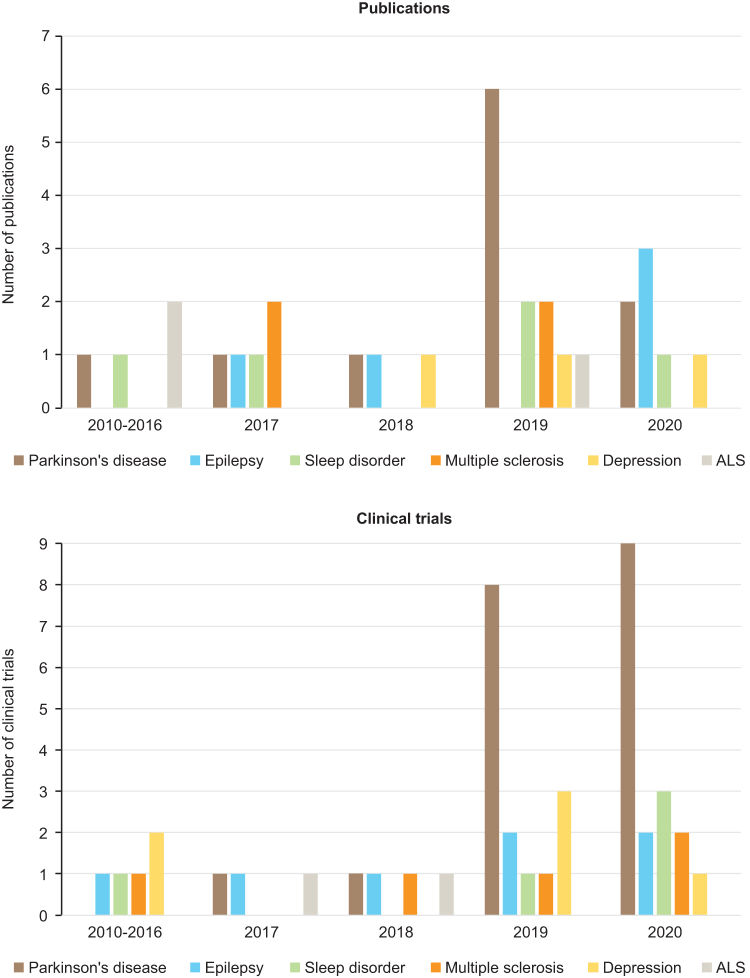
Number of publications and active or actively clinical trials included in this review by date of publication or date of first posting of the clinical trial in a clinical trial database.

This review is focused only on the disorders for which three or more publications were available: Parkinson's disease, epilepsy, sleep disorders, multiple sclerosis, depression, and ALS. In general, most publications and clinical trials for these disorders were very recent and were reported in 2019 or 2020 (*Fig. 3*). The main objectives of the included studies were to quantify symptoms monitored by a digital device or to assess the correlation between conventional clinical evaluations and a digital device (*Table 1*). Very few studies assessed the integration of remote patient monitoring with telemedicine; only three publications^13–15^ and three clinical trials^16–18^ assessed or were assessing the effectiveness of integrating a digital device with telemedicine.

**Table 1.** Literature and Clinical Studies on Remote Patient Monitoring in Neuropsychiatric Disorders

DISORDER	PUBLICATIONS		CLINICAL TRIALS	
	***N***	OBJECTIVES AND INTENDED USE (NUMBER OF PUBLICATIONS)	***N***	STUDY OBJECTIVES (NUMBER OF TRIALS)
Parkinson's disease	11	Symptom quantification using digital devices (5)^20,23,26–28^ Correlation of data from digital devices with conventional clinical assessment (4)^19,21,22,25^ Evaluation of the tolerability of prolonged use of wearable devices (2)^24,29^	19	Symptom quantification using digital devices (15)^17,18,30–33,35–38,40,41,43,44,46^ Auditory or tactile cues for symptom improvement or sudden event detection (5)^33,34,39,42,45^ Comparison of data from digital devices with conventional clinical assessment (2)^31,35^ Correlation between symptom progression and biomarkers (imaging and genetic) (1)^30^ Symptom monitoring for drug planning (1)^18^ Digital device data combined with an online platform for disease management (1)^17^
Epilepsy	5	Development of wearable EEG devices (4)^48–51^ Comparison between clinical and commercial EEG devices (1)^47^	7	Predictors of seizures (3)^45,53,55^ Caregiver warning of anticipated epileptic seizure (1)^56^ Comparison between a clinical and commercial EEG device (1)^54^ Disease management by telemedicine (1)^16^ Monitoring movement to encourage physical activity (1)^52^
Sleep disorders	5	Quantification of sleep parameters using a digital device and correlation with conventional clinical assessment (3)^57–59^ Delivery of behavioral therapies using a smartphone application and a wearable sleep tracking device (2)^13,14^	5	Evaluation of intervention effect by quantification of sleep parameters (3)^60,61,63^ Quantification of sleep parameters using a digital device (1)^62^ Comparison with conventional clinical assessment (1)^64^
Multiple sclerosis	4	Correlation of data from digital devices with conventional clinical assessment (3)^66–68^ Symptom quantification using a digital device (1)^65^	5	Assessment of intervention effect by symptom quantification (5)^69–73^
Depression	3	Correlation of data from digital devices with conventional clinical assessment (2)^75,76^ Assessment of adherence to self-monitoring (1)^74^	6	Assessment of intervention effect by symptom quantification (2)^79,80^ Symptom quantification (1)^82^ Evaluate internet group-based social support (1)^81^ Effectiveness of a smartwatch application as an intervention (1)^77^ Effectiveness of a smartwatch application as an intervention, including internet-based review of symptoms and feedback by a therapist (1)^78^
ALS	3	Comparison of data from digital devices with conventional clinical assessment (2)^83,84^ Disease management using digital device data combined with telemedicine (1)^15^	2	Symptom quantification (1)^85^ Disease management using digital device data (1)^86^

ALS, amyotrophic lateral sclerosis; EEG, electroencephalogram.

### PARKINSON'S DISEASE

There were 11 publications^19–29^ and 19 clinical trials^17,18,30–46^ on the development of digital devices for remote patient monitoring in Parkinson's disease (*Table 1*). Studies on Parkinson's disease have been published every year since 2016, with a maximum of six reports in 2019, and a total of 17 active or actively recruiting trials were first posted in 2019 or 2020 (*Fig. 3*). Of the 11 publications, most were focused on the quantification of specific motor symptoms or functions using a wearable device on various body locations, and five^23–25,28,29^ were conducted to assess the use of or feasibility of using digital devices in remote settings (*Table 1; Supplementary Table S3*). There was no publication that assessed the effectiveness of integrating a digital device with telemedicine. One clinical trial^17^ was designed to assess patient satisfaction with the integration of remote patient monitoring with telemedicine (*Supplementary Table S4*).

Most devices used indices comprising several motion and joint movement parameters to monitor dyskinesia, tremor, gait impairments, and postural instability.^19–21,25–27^ One study reported on the combined use of electrocardiographic parameters, voice patterns, and kinematics to monitor changes in motor symptoms and cognitive function.^22^ One study assessed the use of a wearable accelerometer and barometer to predict falls in a home-based setting,^23^ and one measured muscle contraction and gait frequency to detect freezing of gait in real time.^28^ In two studies, digital indices comprising multiple parameters were analyzed by statistical methods or machine learning before and after treatment to determine whether changes in these indices could predict response to treatment.^20,27^

Four publications examined the correlation between objective digital indices obtained from a wearable device and subjective clinical assessments.^19,21,22,25^ Findings from two of these studies showed strong correlation between objective data from a wearable motion capture system and subjective measures of dyskinesia (Unified Dyskinesia Rating Scale) and tremor (Unified Disease Rating Scale), respectively.^19,21^ Two publications assessed the ability of patients to tolerate a wearable digital device; patients wore the devices at home for 1 month^24^ or 1 week.^29^ In both studies, patients found the body-worn devices to be acceptable and expressed a preference for the devices over manual self-reporting methods such as symptom diaries.

Of the 19 clinical trials that were active or actively recruiting, 15 were designed to quantify symptoms and/or functions.^17,18,30–33,35–41,43,44,46^ The other five studies were designed to assess the use of auditory or tactile cues to facilitate the detection, notification, and prevention of symptoms such as freezing of gait and difficulties with swallowing.^33,34,39,42,45^ While most studies were focused on objective measures for detecting specific motor symptoms or events, there appeared to be growing interest in the use of digital devices for remote disease monitoring,^30,33^ optimization of drug dosing,^18^ and disease management.^17^

The Care-For-One observational study^17^ is designed to assess patient satisfaction with a digital platform for online medical consultations, which includes remote symptom monitoring with digital devices, and patient satisfaction with online instructions provided by a pharmacist on drug use and drug delivery. In this study, information on patient-reported motor and nonmotor symptoms will be collected through a digital device worn at home for 8 weeks, and physicians and pharmacists will conduct their examinations and provide instructions online in accordance with routine clinical practice.

### EPILEPSY

There were five publications^47–51^ and seven clinical trials^16,45,52–56^ on the development of new wearable or portable electroencephalograms (EEGs) for the monitoring of patients with epilepsy (*Table 1*). Publications and active or actively recruiting clinical trials on devices for monitoring patients with epilepsy have emerged increasingly since 2017 (*Fig. 3*). Many of the devices were designed to be worn on the head. One clinical trial was assessing the effectiveness of telemedicine combined with a digital device for patient monitoring.^16^

Four publications were focused on development of wearable head-mounted caps or headsets,^47,49–51^ and one reported on an in-ear EEG, which is likely to improve usability.^48^ Of these, one device intended for inpatient and outpatient use included a detection and warning system for seizure events,^51^ and one portable EEG device was under development for bedside use, which also incorporated wearable functional near-infrared spectroscopy to support measurement of bilateral hemodynamic activity.^50^ One small study compared the diagnostic performance of several commercially available wearable EEGs with video EEG, which is conducted in hospital settings, and simultaneously displays a video recording of patients with their EEG readings. This study showed that the commercial EEG devices had sufficient performance to be used as a screening tool for epilepsy.^47^

In contrast to the publications, which were focused on device development, most active or actively recruiting clinical trials were focused on identification of factors that can predict seizures with a wearable device^45,53,55^ or on development of a device that can notify caregivers of an anticipated seizure^56^ (*Table 1*). One clinical trial assessed whether a wearable device for monitoring patient movement could be used to encourage physical activity in patients with epilepsy.^52^

The clinical trial assessing the integration of telemedicine with remote patient monitoring is designed to assess the effectiveness of video consultations combined with a mobile internet device for monitoring serum levels of antiepileptic drugs compared with routine clinical assessment.^16^ The primary outcome is seizure control, and secondary outcomes include adherence to treatment, adverse events, quality of life, and the occurrence of mood disorders.

### SLEEP DISORDERS

There were five publications^13,14,57–59^ and five clinical trials^60–64^ on the use of wearable or portable devices and/or smartphone applications for use in patients with sleep disorders (*Table 1*). Publications and active or actively recruiting clinical trials on devices for monitoring patients with sleep disorders emerged in 2014 with most becoming available from 2019 (*Fig. 3*). Almost all studies measured sleep indices with devices designed to be worn on the wrist.^13,14,57–64^ Two publications assessed the effectiveness of integrating a digital device with telemedicine.^13,14^

Of the five publications retrieved, three evaluated objective measures of sleep outcomes using a wearable device and correlated these with conventional clinical methods such as patient-reported sleep ratings^57,58^ and inpatient polysomnography.^59^ Two publications assessing integration of telemedicine with remote patient monitoring evaluated the delivery of conventional behavioral therapies through a digital device on sleep outcomes.^13,14^ Both studies incorporated a wearable sleep tracker to monitor activity/sleep integrated with a smartphone application designed to deliver self-help content^13,14^ and telephone coaching.^13^ These studies demonstrated the feasibility of delivering technology-assisted interventions combined with digital monitoring of sleep for improving sleep outcomes.

Of the other publications, one study correlated objective sleep parameters based on wrist-worn actigraphy with patient-reported sleep outcomes^58^ and two studies correlated objective measures of sleep disordered breathing with subjective sleep symptoms (wrist-worn peripheral tonometry)^57^ or conventional polysomnography (wrist-worn reflective photoplethysmography).^59^

Of the five clinical trials that were active or actively recruiting, one was designed to evaluate quantification of sleep parameters using a wearable device,^62^ and three were evaluating the effect of various interventions aimed at improving sleep (e.g., presleep activities/light exposure) using sleep parameters measured by a wearable device.^60,61,63^ One clinical trial was designed to compare sleep activity and patterns measured at home using portable EEG/electrocardiogram (ECG) devices and a wrist accelerometer with conventional inpatient sleep tests (nocturnal polysomnogram).^64^

### MULTIPLE SCLEROSIS

There were four publications^65–68^ and five clinical trials^69–73^ on the development of digital devices for remote patient monitoring in multiple sclerosis (*Table 1*). Studies on devices for remote patient monitoring in multiple sclerosis were published in 2017 and 2019, and clinical trials were active or actively recruiting from 2016 (*Fig. 3*). Most publications and clinical trials assessed physical activity through accelerometers worn on the wrist^65,67–73^ or through a global positioning system (GPS).^66^ There was no publication or clinical trial assessing the effectiveness of integrating a digital device with telemedicine.

Of the four publications retrieved, three correlated objective measures of physical activity measured at home with conventional clinical methods to assess patient disability,^66–68^ and one assessed correlation between physical activity measured at home using an accelerometer and symptoms of fatigue, mood, pain, and cognitive function.^65^ One study compared patients' walking ability using a GPS wristwatch with patients' and neurologists' subjective estimates of walking ability,^66^ and two studies conducted a long-term assessment of step count using a wrist-worn accelerometer and compared the outcomes with step count/activity assessed using actigraphy, manual measurement, and subjective assessment.^67,68^

Of the five clinical trials that were active or actively recruiting, all were designed to verify the effect of various interventions for improving symptoms of multiple sclerosis and included an assessment of physical activity measured through accelerometers. Interventions included the effects of diet,^73^ behavioral programs to improve physical activity and weight,^69–71^ and cognitive behavioral therapy and drug treatment.^72^

### DEPRESSION

There were three publications^74–76^ and six clinical trials^77–82^ on the development of digital devices for remote monitoring of patients with depression (*Table 1*). Publications and most clinical trials were reported consistently from 2018 onward (*Fig. 3*). All publications and most active or actively recruiting clinical trials assessed physical activity and/or sleep, heart rate, and ambient light exposure using commercially available wrist-worn devices. No publication or clinical trial was retrieved that assessed or were assessing the effectiveness of integrating a digital device with telemedicine.

Of the three publications retrieved, two aimed to correlate objective measures of physical activity collected remotely with conventional patient-reported scales of depression symptoms.^75,76^ Findings from these studies suggested that objective measures of physical activity using wearable devices correlate well with subjective evaluations such as the self-reported Beck Depression Inventory-II (BDI-II) and Hamilton Depression Inventory (HAM-D) in patients with major depressive disorder and the Depression Anxiety Stress Scale-21 items in young adults with psychological distress.

One study compared the preference and adherence of patients with bipolar disorder for self-monitoring of physical activity using a smartphone application versus objective monitoring using a wearable activity tracker.^74^ This study showed that adherence to the activity tracker decreased over time and that patients preferred to review their recorded symptoms with a clinician. There was no report on the evaluation of mood symptoms using wearable devices.

Of the six clinical trials that were active or actively recruiting, most were evaluating the effectiveness of behavioral therapies or support systems using various measures that included wearable physical activity or sleep trackers (*Table 1*). One study was designed to develop an objective indicator of physical activity using a wearable device,^82^ and two studies assessed wearable devices to quantify symptoms in response to behavioral therapies.^79,80^ Two studies were evaluating behavioral therapy delivered by a smartwatch application,^77,78^ and one study was evaluating a group-based lifestyle intervention supported by mobile health technology and social media.^81^ Of these, one study was designed to include a review of symptoms by a therapist and feedback by the smartwatch application.^78^

### AMYOTROPHIC LATERAL SCLEROSIS

There were three publications^15,83,84^ and two clinical trials^85,86^ on the development of digital devices for remote symptom monitoring in patients with ALS (*Table 1*). Most publications and active or actively recruiting clinical trials were reported between 2016 and 2019 (*Fig. 3*). The location of wearable devices varied with the outcomes to be assessed. One publication assessed the feasibility of integrating a digital device with telemedicine.^15^

Of the three publications, one was a small study that piloted the use of a portable pulse oximeter, digital transmitter, and remote nurse, who was available for real-time assessment and consultation and who was supported by respiratory physicians, neurologists, and psychologists.^15^ Patients were given a portable oximeter for use during follow-up telephone consultations or, in severe cases, an oximeter and modem to allow transmission of a pulse arterial saturation trace to the remote nurse. Of the 2,224 calls, 260 unscheduled calls were received from patients or caregivers on symptoms and transmission associated with pulse-oximetry data. These calls resulted in two immediate hospital admissions due to severe oxygen desaturation and 80 medical prescriptions in response to changes in oxygen saturation.

Findings from this study showed that home monitoring of oxygen saturation combined with remote nurse-assisted care was feasible for disease management for patients with ALS in their homes. A second publication reported on the ability of a commercially available chest-worn accelerometer and ECG sensor integrated with a digital platform for the remote collection of data on physical activity and heart rate,^83^ and a third publication reported on a study that measured keyboard typing ability using a finger-worn accelerometer as a measure of upper limb dysfunction.^84^

Of the two clinical trials that were active or actively recruiting, one was assessing the feasibility of a portable device for home-based monitoring of expired carbon dioxide as an indicator of breathing failure,^86^ and one was assessing the feasibility of several home-based measures, including respiratory function and physical activity.^85^

## Discussion

This literature review has shown that wearable devices for daily symptom monitoring have been or are currently being developed for several neuropsychiatric disorders, particularly Parkinson's disease. Most studies were focused on kinematic parameters and physical activity for the assessment of motor symptoms or as an indirect measure of mood or sleep. There were no wearable devices for the direct evaluation of psychiatric symptoms and very few for the direct evaluation of nonmotor symptoms. The focus on motor symptoms and physical activity is not unexpected because motor function is seriously affected in many patients with neurological and psychiatric disorders,^5^ but it is hoped that wearable devices for evaluation of cognitive and psychiatric symptoms, as well as other newly emerging technologies such as the in-ear EEG, will receive similar focus in the future.

Although several studies have validated the use of wearable devices against gold-standard clinical measures and subjective patient-reported outcomes, the real-world benefits of these devices in terms of improving clinical outcomes and patient prognoses, improving the quality of life of patients and carers, and achieving long-term patient acceptance and adherence are not yet known. In addition, models for implementing remote patient monitoring in telehealth settings for chronic neurological diseases have not been fully developed.

### PARKINSON'S DISEASE

Parkinson's disease is a complex disorder in which patients experience a highly heterogeneous range of motor and nonmotor symptoms that appear and worsen over many years.^87^ The most common motor symptoms that contribute to patient disability are tremor, bradykinesia, muscle rigidity, and impaired posture, balance, and gait. Personalized management that focuses on controlling motor and nonmotor symptoms is recommended,^88,89^ but monitoring the changes in severity of individual symptoms is challenging, and conventional rating scales can be limited in their ability to detect subtle changes in severity or discriminate the effects of treatment on specific symptoms.^12,90^

This literature review has shown that there is a continued interest in the development and use of objective digital indicators of symptom severity for patients with Parkinson's disease. These digital indicators have the potential to contribute to the assessment of treatment effectiveness and to be integrated into health care delivery with smartphone applications and other response cues to facilitate real-time improvement in symptoms. Currently, development of objective digital indices is primarily focused on measuring changes in motor symptom severity and ranges from assessments of gait frequency and fall rates to complex algorithms comprising multiple motion and kinematic parameters (*Table 2*). Relatively few studies have correlated these indices with conventional symptomatic rating scales or have assessed the efficacy of remote digital monitoring in home-based settings.

**Table 2.** Current Trends and Future Perspectives

DISEASE AREA	CURRENT TRENDS	FUTURE PERSPECTIVES AND THE ROLE OF TELEMEDICINE
Parkinson's disease	Current research is focused on development of objective indices of symptoms using wearable digital devices and the use of these indices for assessing efficacy of interventions Most studies are developing objective indices of motor symptoms using a combination of movement and kinematic parameters and are using the indices to measure changes in symptom severity Studies are assessing the correlation of objective indices with conventional clinical rating scales and are confirming their efficacy	It is anticipated that wearable devices will provide objective indices of Parkinson's disease symptoms Because of the complexity of Parkinson's disease symptomatology, continuous measurement of symptoms in daily life will facilitate the identification and prediction of changes in symptom severity for individual patients This information will be instrumental in tailoring treatment to individual patient needs, thereby making it possible to provide optimal treatment
Epilepsy	Current research is focused on the use of wearable EEG devices for the detection and notification of seizures Non-EEG objective indices for detection of predictors of seizures (movement, sweating, and heart rate) and comorbidities such as stroke (e.g., hemodynamic activity) are also being developed The use of wearable digital devices for antiepileptic drug monitoring is being investigated	It is anticipated that wearable EEG devices will be used for diagnosis of epilepsy in the future Measurement of brain activity by EEG in daily life is feasible and will reduce patient burden associated with video EEG conducted in clinical settings Continuous and long-term measurement of EEG will improve seizure detection, prediction, and treatment for individual patients Devices that incorporate automatic notification of seizures to clinicians and caregivers will help alleviate caregiver burden
Sleep disorder	Current research is focused on the development of objective indices of symptoms using wearable digital devices and the use of these indices for assessing efficacy of interventions Studies have assessed objective indices (apnea-hypopnea index, total sleep time, and sleep efficiency) for changes in sleep symptoms The correlation between indices obtained from device data with gold-standard polysomnography and subjective patient-reported measures is being investigated Integration of technology-assisted interventions with digital sleep monitoring is being assessed	It is anticipated that objective indices from wearable devices will be used for remote monitoring of sleep parameters Accurate sleep monitoring at home will facilitate diagnosis, long-term symptom monitoring, and appropriate treatment Clinicians who receive information on home-based sleep management and interventions can work closely with patients to facilitate patient self-management
Multiple sclerosis	There is a high level of interest in developing wearable devices for objective indices of physical activity and fatigue The current focus is on correlating objective indices against manual measures of physical activity and subjective assessment of disability and using these indices to measure efficacy of behavioral interventions and drug treatment	It is anticipated that objective indices from wearable devices will be used for remote monitoring of multiple sclerosis symptoms Current subjective methods for assessment of disability are less accurate than objective indices and may not be adequate for assessing changes in symptoms or symptom severity By acquiring long-term data in daily life, it will be possible to gain a deeper understanding of patient symptoms and disability and provide individualized treatment programs
Depression	Current research is focused on the development of objective indices of symptoms using wearable digital devices and the use of these indices for assessing efficacy of interventions Most reports use commercially available devices and physical activity indices to measure symptom changes Correlation between objective indices and subjective patient-reported measures of depression symptoms is being investigated There is an increased interest in remote delivery of behavioral interventions using smartwatch applications	It is anticipated that objective indices of physical activity from wearable devices will be used for remote detection of changes in depression symptoms For patients with depression, regular monitoring is essential to detect worsening of symptoms, but repeated clinical evaluation is necessary, which contributes to substantial burden Real-time monitoring with devices provides a low-burden, step-by-step picture of mood changes, allowing for the detection of sudden worsening of symptoms and early intervention
ALS	Current research is focused on the development of objective indices of ALS symptoms Studies are assessing the use of objective indices of physical activity, motor skills, and respiratory function to understand disease progression and improve disease management One study assessed the feasibility of integration of digital measurement of respiratory function at home with telemedicine	It is anticipated that various objective indices will be used for remote monitoring of ALS symptoms ALS is associated with significant disability and requires long-term monitoring, care, and cooperation with clinicians, which place a large burden on caregivers Use of digital objective indices for ALS symptoms in telemedicine will enable home-based symptom monitoring and disease management that will reduce caregiver burden

ALS, amyotrophic lateral sclerosis; EEG, electroencephalogram.

Because wearable devices have the capacity to enable real-time monitoring of patient symptoms, they have the potential to be used in telehealth settings and will allow the development of improved and individualized medical care for all patients with Parkinson's disease, irrespective of whether they experience obstacles accessing inpatient care. However, there were no studies currently assessing integration of a wearable device with telemedicine, and future studies are needed to evaluate the use of wearable digital devices for disease management in these remote settings.

### EPILEPSY

Diagnosis of epilepsy typically requires an episodic record of seizures from the patient and/or witness, and continuous monitoring by EEG in inpatient settings.^91^ Ambulatory EEGs have emerged as less expensive and less burdensome methods for the detection and quantification of seizures that may improve diagnosis and management of patients with epilepsy.^92^

Current research is focused on the use of wearable EEGs for the detection and notification of seizures, and other non-EEG indicators are under development to enable the prediction of seizures and the detection of comorbidities such as stroke (*Table 2*). Continuous and long-term measurement of brain activity by a wearable or portable EEG device have a role in disease management as it has the potential to improve seizure detection, prediction, and treatment for individual patients and will relieve patients of the burden of inpatient EEG measurement. Furthermore, the ability to incorporate an early warning system or notification of seizures will help alleviate caregiver burden in the very near future.

### SLEEP DISORDERS

Sleep disorders comprise a variety of diseases, which include sleep-disordered breathing (e.g., snoring and obstructive sleep apnea), difficulties initiating or maintaining sleep (e.g., insomnia), or excessive daytime sleepiness (e.g., narcolepsy). The gold-standard measure for sleep disorders is polysomnography, which requires an overnight stay in a sleep clinic.^93^ Wearable movement trackers that monitor physical activity and sleep patterns are recommended over sleep diaries for the assessment of symptom severity in daily practice.^94^ In addition, because of the impact of poor sleep on patient quality of life in many disease settings, sleep-related patient-reported outcomes such as those in the MD Anderson Symptom Inventory are often incorporated into clinical practice to monitor symptom severity and the effects of sleep symptoms on patients' quality of life.^11^

Because of the need for long-term sleep assessment in patients with sleep disorders, there is growing interest in the use of wearable devices that can provide an accurate and objective measure of sleep quality at home (*Table 2*). The main focus of current research is the development of objective indices for measuring changes in symptom severity and for confirming the effectiveness of objective indices compared with polysomnography and with subjective patient-reported outcomes. It is anticipated that objective indices from wearable devices will be used for remote monitoring and management of sleep symptoms, particularly those associated with chronic conditions such as obstructive sleep apnea, which has long-term consequences for patient health. Accurate sleep monitoring at home with a wearable device will enable diagnosis and long-term symptom monitoring and can be integrated into telemedicine settings for the delivery of appropriate treatment by both clinicians and patients.

### MULTIPLE SCLEROSIS

Multiple sclerosis is primarily associated with impaired motor symptoms that worsen with time and contribute to significant disability. In addition, patients experience cognitive impairment, pain, depressed mood, and fatigue. Because patients are at high risk of a sedentary lifestyle and low quality of life, many behavioral interventions are focused on improving physical activity.^95,96^ Currently, patient-reported outcome measures such as the Expanded Disability Status Scale are widely used in clinical practice to monitor disease progression and changes in disability.^10^

Findings from this literature review showed that the main focus of research is on the development of wearable devices that can provide an accurate and objective measure of physical activity at home (*Table 2*). These objective measures are currently in use to assess the efficacy of interventions and can provide a more accurate assessment of physical activity and disease progression than subjective patient-reported outcomes. The use of wearable digital devices for physical activity is being integrated with other online or digital technologies to deliver remote health interventions. By acquiring long-term data in daily life, it will be possible to gain a deeper understanding of patient symptoms and disability and provide individualized treatment programs.

### DEPRESSION

Assessment of patients with depressive symptoms is mostly subjective and relies on patient self-reports of mood symptoms using well-established tools such as the BDI-II and HAM-D, clinical evaluation, and observations from other health care providers and caregivers.^9^ Routine monitoring of patient symptoms is vitally important for detecting symptom deterioration and for ensuring that patients receive timely interventions. As such, there has been an increased interest in the use of digital devices or internet-based tools to support the monitoring of symptoms and delivery of health care interventions for patients with mental illness.^97^

Currently, research is focused on the delivery of remote health care interventions through internet-based programs and smartphone applications and the use of objective measures of physical activity using commercially available wearable devices to assess the effectiveness of these interventions (*Table 2*). Some studies have attempted to correlate changes in physical activity with changes in subjective patient-reported symptoms, and it is feasible that real-time remote monitoring of physical activity and sleep with a digital device will allow detailed day-to-day information on potential changes in mood and reduce the burden associated with inpatient monitoring of sleep parameters. Therefore, information gained from real-time monitoring is likely to be a valuable resource that can assist health care providers in delivering early interventions, optimizing drug dose levels in a timely manner, and encouraging patient engagement in their health care plans.

However, it is highly likely that the trends identified for depression are also applicable to other mental health conditions. This is especially the case for dementia and schizophrenia where new telephone- and video-based screening and assessment tools have already been validated for use in telemedicine settings^98–100^ and where patients may have comorbid behavioral and psychological symptoms, including depression, anxiety, and sleep disorder.^101–103^ In this study, we identified two peer-reviewed publications^104,105^ and eight active or actively recruiting clinical trials^106–113^ on the use of digital devices for patients with cognitive impairment.

Most studies were focused on the use of digital devices for assessing improvement in cognitive health through behavioral interventions and/or quantification of physical activity and sleep rather than on measurement of cognitive impairment *per se*. The single peer-reviewed publication^114^ on schizophrenia that was retrieved in our search suggests that digital devices may also be used in telemedicine settings for the assessment of disease severity through measurement of autonomic symptoms, including heart rate, electrodermal activity, and physical activity.

### AMYOTROPHIC LATERAL SCLEROSIS

ALS is a motor neuron disease characterized by rapid progression, deterioration in muscle strength, difficulties with swallowing and communication, and fatigue. Prognosis is poor and most patients die of respiratory failure within 3 to 5 years of experiencing their first symptoms. As such, supportive care at home requires a highly multidisciplinary team of health care professionals and incurs substantial burden on caregivers. Current research is focused on the development of objective indices of physical activity, motor skills, and respiratory function, which will assist in remote monitoring of disease progression (*Table 2*). Because ALS is associated with significant disability and requires long-term care, it is anticipated that caregiver burden can be reduced by the use of remote digital monitoring of symptoms that is integrated into telehealth settings.

This literature review has provided a comprehensive assessment of the status of research for remote patient monitoring for neurological and psychiatric diseases. A key feature of this review is that it was designed to capture recently published studies as well as active or actively recruiting clinical trials to identify future trends.

The main limitations of this literature review are (1) the literature searches were not systematic and focused on peer-reviewed publications in English and Japanese, and clinical trials from Japan, Europe, the United Kingdom, and the United States only, (2) because the focus was on most recent and future trends, we only included information on those diseases with three or more peer-reviewed publications and we did not include information summarized in other review articles, which may have contributed to selection bias, (3) the quality of the information on each of the clinical trials was limited to what was reported in each database, and (4) unregistered observational studies that may have been ongoing were not identified.

## Conclusions

The emergence of COVID-19 has thrust telemedicine to the forefront of health systems research and has shown that quality health care can be administered remotely.^3,4^ For patients with chronic neurological diseases who have limited access to specialist care, the use of wearable devices that provide objective information about disease progression and treatment effectiveness is likely to transform the way in which patients and their carers engage with health care systems.

Current studies suggest that longitudinal measurement of data on a daily basis can provide clinicians and carers with the information needed to optimize and individualize treatment and can potentially enable early detection and/or prevention of acute symptom deterioration or adverse events. Once telemedicine with remote patient monitoring is implemented, patients and caregivers are likely to experience reductions in the burden associated with treatment of chronic neurological and neurodegenerative diseases and improvements in quality of life.

However, evidence that digital devices can be integrated successfully into telehealth settings or lead to improved outcomes for patients with neuropsychiatric disorders who require long-term, home-based care is limited. Because the potential benefits of telemedicine to patients, carers, and societies are substantial, further research is needed to advance the use of new technologies for remote patient monitoring and health care delivery.

## Data Sharing

Data sharing is not applicable to this article as no datasets were generated or analyzed during this study.

## Supplementary Material

Supplemental data

Supplemental data

Supplemental data

Supplemental data

## Authors' Information

All authors participated in the conceptualization of the study, and in the drafting, critical revision, and approval of the final version of the article. In addition, T.S. was involved in data curation, formal analysis, investigation, supervision of the study, and preparation of the first draft; Y.F. was involved in the data curation, formal analysis, investigation, methodology, supervision of the study, and preparation of the first draft; A.H. was involved in the data curation, formal analysis, investigation, methodology, project administration, and preparation of the first draft, and was responsible for resources and software; M.O. was involved in supervision of the study; and J.F. was involved in funding acquisition and supervision of the study.
