# Supplementary material for: Remote Patient Monitoring for Neuropsychiatric Disorders: A Scoping Review of Current Trends and Future Perspectives from Recent Publications and Upcoming Clinical Trials
Source: Telemed J E Health. 2022 Sep 7;28(9):1235–50. doi: 10.1089/tmj.2021.0489 (PMC9508442; doi:10.1089/tmj.2021.0489)
Supplement: Supplemental data [file Suppl_TableS1.docx]

**Table S1. Search hierarchy and terms for the literature databases**

|  | **Search terms** | |
| --- | --- | --- |
| **Search hierarchy** | **PubMed** | **ICHUSHI** |
| Wearable, Home, Portable | “Wearable Electronic Devices“ [MeSH] OR “home monitoring“ [ti,ab] OR “portable device“ [ti,ab] | [wearable electronics]/TH OR “home monitoring”/TA OR “portable device”/TA OR Home Monitoring/TA OR [personal digital assistant]/TH |
| Neurological disease,  Psychiatric disorders | “Nervous System Diseases“ [MeSH] OR “Central Nervous System Diseases“ [MeSH] OR “Biological Psychiatry“ [MeSH] OR “Psychiatry“ [MeSH] OR “Preventive Psychiatry“ [MeSH] | [Nervous system disorders]/TH OR [Central nervous system disorders]/TH OR [biological psychiatry]/TH OR [Psychiatry]/TH OR [preventive psychiatry]/TH OR [Psychiatric disorders]/TH |
| Remote device related | (“remote*“ [ti,ab] OR "ONLINE“ [ti,ab] OR “digital“ [ti,ab] OR “tele*" [ti,ab] OR “mobile” [ti,ab] OR “ambulatory” [ti,ab] OR “wrist-worn“ [ti,ab]) AND  (“device“ [ti,ab] OR “application“ [ti,ab] OR “equipment“ [ti,ab] OR “system” [ti,ab] OR “monitor*“ [ti,ab] OR “sensor” [ti,ab] OR “solution*” [ti,ab]) | (“remote”/TA OR “ONLINE”/TA OR “digital”/TA OR “TELE”/TA OR “mobile”/TA OR Remote/TA OR Online/TA OR Digital/TA OR Remote/TA OR Mobile/TA AND  (“device”/TA OR “application”/TA OR “EQUIPMENT”/TA OR “System”/TA OR “monitor”/TA OR “sensor”/TA OR “solution”/TA OR Device/TA OR Application/TA OR Device/TA OR System/TA OR Monitor/TA OR Sensor/TA OR Resolution/TA) |
| Remote medical care | “Telemedicine“ [MeSH] OR “Telemetry“ [MeSH] OR “Telehealth“ [ti,ab] OR “Tele*“ [ti,ab] | [telemedicine]/TH OR [telemetry]/TH OR “telehealth”/TA OR Distant/TA |

MeSH, Medical Subject Headings; TA, title/abstract; TH, thesaurus term; ti,ab, title/abstract.
* denotes truncation
