# Supplementary material for: Remote Patient Monitoring for Neuropsychiatric Disorders: A Scoping Review of Current Trends and Future Perspectives from Recent Publications and Upcoming Clinical Trials
Source: Telemed J E Health. 2022 Sep 7;28(9):1235–50. doi: 10.1089/tmj.2021.0489 (PMC9508442; doi:10.1089/tmj.2021.0489)
Supplement: Supplemental data [file Suppl_TableS2.docx]

**Table S2. Search hierarchy and terms for clinical trial databases**

| **Search hierarchy** | **Search expression** |
| --- | --- |
| Wearable, Home, Portable | wearable OR home OR portable |
| Disorder | Parkinson Disease OR Epilepsy OR Multiple sclerosis OR Depression OR Dyssomnia OR Amyotrophic lateral sclerosis OR Dementia |
| Device-related monitoring | application OR equipment OR device OR system OR solution OR monitor OR sensor |
| Remote-related | remote OR online OR digital OR tele OR mobile OR smart phone OR ambulatory OR wrist-worn |
| Region | Region (US, Europe, United Kingdom, and Japan) |
| Remote medical care | telemetry OR telehealth OR telemedicine |
