# Supplementary material for: Remote Patient Monitoring for Neuropsychiatric Disorders: A Scoping Review of Current Trends and Future Perspectives from Recent Publications and Upcoming Clinical Trials
Source: Telemed J E Health. 2022 Sep 7;28(9):1235–50. doi: 10.1089/tmj.2021.0489 (PMC9508442; doi:10.1089/tmj.2021.0489)
Supplement: Supplemental data [file Suppl_TableS3.docx]

**Table S3. Summary of publications retrieved from the literature search**

| **Study** | **Objective** | | | | **Device type** | | | **Device name(s)** | | | **Device output** | | | **Integration with telemedicine** | |
| --- | --- | --- | --- | --- | --- | --- | --- | --- | --- | --- | --- | --- | --- | --- | --- |
| **Parkinson’s disease** | | |  | | | |  | | |  | | |  | | |
| Fisher et al. 2016^29^ | Evaluation of the tolerability of long-term wearing of wearable devices | | | | Wearable accelerometer (both wrists) | | | AX3 (Axivity Ltd) | | | Kinematic parameters | | | No | |
| Delrobaei et al. 2017^19^ | To develop an objective measure of dyskinesia using wearable technology | | | | Wearable motion capture system with sensing units (full body) | | | IGS-180 (Synertial Ltd) | | | Kinematic parameters | | | No | |
| Delrobaei et al. 2018^21^ | To compare objective measures of dyskinesia with wearable devices with conventional clinical assessments | | | | Wearable motion capture system with sensing units (full body) | | | IGS-180 (Synertial Ltd) | | | Kinematic parameters | | | No | |
| Botres et al 2019 | Assessment of device tolerability and adherence | | | | Wearable accelerometer (wrist, ankle, hip) and ambient room and door sensors | | | AX3 (Axivity Ltd)  PIR sensors  DomoCare (DomoSafety Ltd) | | | Kinematic parameters  Patient movement and location | | | No | |
| Ferraris et al. 2019^25^ | To assess the feasibility of home-based automated assessment of postural instability and lower limb impairment | | | | Indoor stationary device with mini-PC and monitor | | | Kinect v2 (Microsoft Inc) | | | Kinematic parameters | | | No | |
| Ghassemi et al. 2019^26^ | To present a new system using wearable devices for analyzing turning anomalies | | | | Wearable movement sensor (each shoe below ankle) | | | Shimmer 2R/3 (Shimmer Inc) | | | Turn-derived parameters | | | No | |
| Khodakarami et al. 2019^27^ | To test whether data from wearable devices can predict response after levodopa administration | | | | Wearable accelerometer (wrist) | | | PKG logger (Global Kinetics Pty Ltd) | | | Kinematic parameters | | | No | |
| Mazzetta et al. 2019^28^ | Development of an algorithm to detect FOG | | | | Wearable motion sensor device (right leg) | | | Bio2Bit Move, experimental | | | Kinematic parameters | | | No | |
| Ryu et al. 2019^22^ | Development of a platform for digitizing clinical symptom assessments | | | | Wearable motion capture system (full body), portable device (head, stomach), microphone | | | Motion capture system  EEG device  Microphone | | | Measurement of cognition and motor control using pen movement, EEG and heart signals, and voice | | | No | |
| Evers et al. 2020^20^ | Evaluation of whether motion fluctuation in real life can be monitored objectively and remotely by gait analysis using a device | | | | Wearable motion sensor (wrist, ankle, back, abdomen), synchronized video filming, smartwatch, smartphone app for assessing disease severity, and physiological sensors | | | Physilog4 (Gait Up SA)  Hopkins PD App (John Hopkins Medicine)  E4 Wristband (Empatica Inc) | | | Kinematic parameters | | | No | |
| Silva et al. 2020^23^ | Quantification of fall incidence in real life using a wearable device | | | | Wearable tri-axial accelerometer and barometer (neck) | | | Personal Emergency Response System (PERS, Philips) | | | Fall events (changes in height, orientation, and impact) | | | No | |
| **Epilepsy** |  | | | |  | | |  | | |  | | |  | |
| Pinho et al. 2017^51^ | Assessment of a novel wearable EEG system for monitoring of epilepsy | | | | Wearable EEG cap (head) | | | EEG cap | | | EEG signals | | | No | |
| Kassab et al. 2018^50^ | Validation and performance of a prototype wearable fNIRS-EEG system for long-term clinical monitoring | | | | Wearable EEG cap (head) | | | Wireless multichannel fNIRS-EEG | | | EEG signals and hemodynamic brain activity | | | No | |
| Carvalho et al. 2020^49^ | To demonstrate the clinical value of sub-spike index quantification and validation of a wearable EEG device | | | | Wearable EEG device (chest and head) | | | Standard scalp disk electrodes | | | EEG signals | | | No | |
| Sintotskiy et al. 2020^48^ | To compare the quality of test signals and noise characteristics of a portable EEG system with a conventional EEG amplifier | | | | Wearable EEG device (ears) | | | In-ear EEG | | | EEG signals | | | No | |
| Titgemeyer et al. 2020^47^ | To compare clinical EEG with a mobile consumer-grade EEG for diagnosis of epilepsy | | | | Wearable EEG headset (head) | | | Emotiv EPOC (Emotiv, Inc) | | | EEG signals | | | No | |
| **Sleep disorders** | | |  | | | |  | | |  | | |  | | |
| Park et al. 2014^57^ | Assessment of the accuracy and efficacy of a wrist-worn device for peripheral arterial tonometry for evaluation of surgical outcomes in patients with obstructive sleep apnea | | | | Wearable peripheral arterial tonometer (wrist and finger) | | | watch-PAT 200 (Itamar Medical Ltd) | | | Respiratory disturbance index, apnea-hypopnea index, lowest oxygen saturation, and valid sleep time | | | No | |
| Kang et al. 2017^14^ | Evaluation of the effectiveness of CBT for insomnia with a mobile application for treating insomnia | | | | Wearable activity tracker (wrist) and smartphone | | | Fitbit Charge HR (Fitbit Inc)  App | | | Total sleep time and sleep efficacy  CBT for insomnia delivered via an app | | | Yes | |
| Baron et al. 2019^13^ | Evaluation of the feasibility of technology-assisted behavior intervention on sleep prolongation | | | | Wearable activity tracker (wrist) and smartphone | | | Actiwatch Spectrum Plus (Philips Respironics, Inc)  App | | | Various sleep parameters | | | Yes | |
| Komarzynski et al. 2019^58^ | To explore objective actigraphy metrics that correlate with subjective sleep ratings | | | | Wearable activity tracker (wrist) | | | Micro Motion Logger (Ambulatory Monitoring Inc) | | | Various sleep parameters using accelerations per 1 min epochs | | | No | |
| Papini et al. 2020^59^ | Assessment of a new wrist-worn method for measuring automated apnea-hypopnea index | | | | Wearable rPPG device (wrist) | | | Wrist-worn rPPG device (Philips) | | | Cardiovascular, respiratory, and sleep activity derived from rPPG signals | | | No | |
| **Multiple sclerosis** | |  | | | |  | | |  | | |  | | |  |
| Block et al. 2017^67^ | Assessment of the association between average daily step count and MS disability | | | | Wearable activity tracker and actigraph (wrist) | | | Fitbit Flex (Fitbit, USA) ActiGraph GT3X (Manufacturing Technology, Inc) | | | Comparison of physical activity (step count monitoring) between commercial activity tracker and research-grade actigraphy | | | No | |
| Dalla-Costa et al.  2017^66^ | Evaluation of concordance between patient and neurologist assessment of walking ability and assessment of walking ability using a wearable device | | | | Wearable GPS smartwatch (wrist) | | | Forerunner 230 (Garmin Ltd) | | | Mean maximum walking ability using walking distance | | | No | |
| Block et al. 2019^68^ | Assessment of the relationship between MS symptoms and step count | | | | Wearable activity tracker (wrist) | | | Fitbit Flex (Fitbit Inc) | | | Physical activity (step count monitoring) | | | No | |
| Kratz et al. 2019^65^ | Assessment of the relationship between physical activity measured using wearable devices and MS symptoms | | | | Wearable activity tracker (wrist) | | | PRO-Diary (CamNTech, Ltd) | | | Physical activity | | | No | |
| **Depression** |  | | | |  | | |  | | |  | | |  | |
| Knight et al. 2018^75^ | Differences in tolerability between wearable devices and smartphone apps in adolescents with psychiatric disorders and whether activity data collected with wearable devices and smartphone apps can help treat psychiatric disorders | | | | Wearable activity tracker (wrist) and smartphone | | | Various commercially available activity trackers and apps | | | Physical activity | | | No | |
| Jacobson et al. 2019^76^ | Evaluate the severity of depression based on biological data | | | | Wearable actigraph and light sensor device (wrist) | | | Actiwatch-L (Philips Respironics, Inc) | | | Physical activity and ambient light exposure in lux every 15 minutes | | | No | |
| Van Til et al. 2020^74^ | Search for ways to enhance the continuity of self-monitoring in patients with bipolar disorder | | | | Wearable activity tracker (wrist) and smartphone | | | Fitbit Alta HR (Fitbit Inc)  App | | | Physical activity, sleep, heart rate  Self-reported symptoms for mania (increased energy, rapid speech, irritability) and depression (depressed mood, fidgeting, fatigue) | | | No | |
| **Amyotrophic lateral sclerosis** | | | |  |  |  |  |  |  |  |  |  |  |  |  |
| Londral et al. 2016^84^ | Verify keyboard activity and identify markers of upper arm weakness | | | | Wearable 3-axis accelerometer (index finger of dominant hand) | | | Experimental | | | Time and acceleration of index finger movement (typing tasks) | | | No | |
| Garcia-Gancedo et al. 2019^83^ | Validate the feasibility of a new digital platform for remote recording of multiple symptoms (physical activity, heart rate variability, and speech) and its impact on patients' daily lives and tolerability | | | | Wearable 3-axis accelerometer, ECG and heartbeat sensor (chest) | | | Mega Faros 180, 2-lead ECG sensor, Mega Fast Fix electronic disposable patch (Mega Electronics Ltd) | | | Physical activity, heart rate variability | | | No | |
| Vitacca et al. 2010^15^ | Verify the feasibility of comprehensive medical care using telemedicine | | | | Wearable pulse oximeter (finger) | | | Onyx 9500 (Nonin Medical Inc) | | | Oxygen levels | | | Yes | |

CBT, cognitive behavioral therapy; ECG, electrocardiogram; EEG, electroencephalogram; fNIRS, functional near-infrared spectroscopy; FOG, freezing of gait; GPS, global positioning system; MS, multiple sclerosis; PD, Parkinson’s disease; PIR, passive infrared; rPPG, remote photoplethysmography.
