# Supplementary material for: Remote Patient Monitoring for Neuropsychiatric Disorders: A Scoping Review of Current Trends and Future Perspectives from Recent Publications and Upcoming Clinical Trials
Source: Telemed J E Health. 2022 Sep 7;28(9):1235–50. doi: 10.1089/tmj.2021.0489 (PMC9508442; doi:10.1089/tmj.2021.0489)
Supplement: Supplemental data [file Suppl_TableS4.docx]

**Table S4. Summary of clinical trials retrieved from the literature search**

| **Title and study identifier** | **First posted** | | **Objective** | | **Device type** | **Device name(s)** | **Device output** | **Target sample size** |
| --- | --- | --- | --- | --- | --- | --- | --- | --- |
| **Parkinson’s disease** |  | |  | |  |  |  |  |
| The Personalized Parkinson Project (PPP)  ClinicalTrials.gov Identifier: NCT03364894^30^ | 2017 | | To create a longitudinal dataset of clinical, molecular, imaging, and continuous wearable sensor-based data from a representative PD cohort | | Wearable activity tracker (wrist) | Study Watch (Verily Life Sciences) | Kinematic parameters, pulse, skin temperature, and ambient conditions | 520^a^ |
| Wearable Assessments in the Clinic and Home in PD (WATCH-PD)  ClinicalTrials.gov Identifier: NCT03681015^31^ | 2018 | | To evaluate disease progression in individuals with early PD, as assessed by digital and electronic sensor data correlated with typical clinical assessments | | Wearable accelerometer and gyroscope | NR | Kinematic parameters | 135 |
| Phase 3 Clinical Effect Durability of TD-9855 for Treating Symptomatic nOH in Subjects With Primary Autonomic Failure (REDWOOD)  ClinicalTrials.gov Identifier: NCT03829657^32^ | 2019 | | To evaluate the sustained benefit in efficacy and safety of ampeloxetine in subjects with primary autonomic failure and symptomatic neurogenic orthostatic hypotension | | Wearable activity tracker | NR | Kinematic parameters | 258 |
| Improving Walking Ability in Parkinson Disease (PASTA)  ClinicalTrials.gov Identifier: NCT03921697^33^ | 2019 | | To develop an application and database for disease management that uses a wearable sensor in combination with voice stimulation to measure motor function for patients with PD | | Wearable sensors | NR | Kinematic parameters | 300 |
| Digital Wearable Walking Aid for Freezing of Gait in Parkinson's Disease  ClinicalTrials.gov Identifier: NCT03978507^34^ | 2019 | | To investigate the effects of relatively long-term use of a wearable device that provides personalized and intelligent cues when freezing of gait is detected | | Foot-mounted sensors, smartphone, earphone | DeFOG system (MHealth Technologies) | Kinematic parameters | 62 |
| Sensor-supported Classification of Gait Patterns in Everyday Movement of Patients With Parkinson's Disease (PD-GPC)  ClinicalTrials.gov Identifier: NCT04054856^35^ | 2019 | | To identify changes in everyday life behavior that correlate with changes in PD symptom indices | | Wearable movement sensors | Integrated Posture and Activity NEtwork by Medit Aachen (IPANEMA) Body Sensor Network (MedIT Aachen) | Kinematic parameters | 50 |
| Monitoring of Mobility of Parkinson's Patients for Therapeutic Purposes - Clinical Trial (MoMoPa-EC)  ClinicalTrials.gov Identifier: NCT04176302^18^ | 2019 | | To assess the clinical effectiveness of the Parkinson’s Holter compared with traditional clinical practice in terms of off-time reduction compared with baseline (recorded using a motor fluctuation diary) | | Wearable accelerometer | STAT-ON^™^ sensor and app (Staton Holter) | Kinematic parameters | 162 |
| Study on levodopa-containing preparation dose adjustment of istradefylline in Parkinson's disease patients (intervention study)  jRCT Identifier: jRCTs031180248^43^ | | 2019 | | To evaluate the efficacy of a therapeutic drug (istradefylline) using acceleration data from a wearable device in patients with PD | Wearable activity tracker | NR | Kinematic parameters, sleep | 111 |
| An observational study for the application of wearable, multi-sensor technology to characterize motor function of Parkinson's disease patients in Japan  JAPIC-CTI Identifier: JapicCTI-194825^46^ | | 2019 | | To use a digital device to characterize motor function in patients with PD | Wearable activity tracker (wrist) | Study Watch (Verily Life Sciences) | Kinematic parameters | 120 |
| Non-invasive AI wearable device for supporting medical care  UMIN Identifier: UMIN000038690^45^ | | 2019 | | To develop an artificial intelligence system that predicts sudden events using a non-invasive wearable device for patients with central nervous system disease | Wearable device | NR | NR | 100 |
| Using Wearable and Mobile Data to Diagnose and Monitor Movement Disorders  ClinicalTrials.gov Identifier: NCT04231487^36^ | | 2020 | | To assess the performance of a smartphone app for estimating symptom severity in patients with movement disorders | Smartphone app | NR | NR | 210 |
| Gait Characteristics and Cognitive Evolution in Parkinson Disease (GECO-PD)  ClinicalTrials.gov Identifier: NCT04297800^37^ | 2020 | | To investigate which gait parameters may be correlated to cognitive impairment (Attention/Executive Function Domain) in a cohort of ambulatory PD patients followed for 3 years | | Wearable triaxial accelerometer, gyroscope, and magnometer | G-walk (Gait and Motion Technology Ltd) | Kinematic parameters | 60 |
| Study Comparing Continuous Subcutaneous Infusion Of ABBV-951 With Oral Carbidopa/Levodopa Tablets For Treatment Of Motor Fluctuations In Adult Participants With Advanced Parkinson's Disease  ClinicalTrials.gov Identifier: NCT04380142^38^ | 2020 | | To assess the efficacy (in terms of a reduction in motor symptom fluctuation), safety, and tolerability of ABBV-951 versus oral levodopa/carbidopa in advanced PD | | Wearable activity tracker | KinetiGraph® (Global Kinetics Pty Ltd) | Kinematic parameters, sleep | 130 |
| Usability of a Novel Cueing Device for Patients With Parkinson's Disease (TCP)  ClinicalTrials.gov Identifier: NCT04459559^39^ | 2020 | | To evaluate the usability of a novel cueing device for patients with PD | | Wearable tactile cueing device | NR | NR | 16 |
| Aquaporin-4 Single Nucleotide Polymorphisms in Patients With Idiopathic and Familial Parkinson's Disease  ClinicalTrials.gov Identifier: NCT04553185^40^ | 2020 | | To understand the relationship between problems in sleep, genetic variations in the aquaporin-4 gene (AQP4), and the development of PD | | Wearable activity tracker | NR | Sleep | 800 |
| Novel Paradigms of Deep Brain Stimulation for Movement Disorders  ClinicalTrials.gov Identifier: NCT04563143^41^ | 2020 | | To better characterize the effects of deep brain stimulation in patients with PD who have undergone surgery for deep brain stimulation by recording data with a wearable accelerometer and tachometer | | Wearable accelerometer and gyroscope | NR | Kinematic parameters | 20 |
| Therapeutic Swallow Sensor  ClinicalTrials.gov Identifier: NCT04664634^42^ | 2020 | | Development and early-stage validation of a wearable sensor for dysphagia in patients with PD | | Wearable sensor for dysphagia | NR | NR | 50 |
| An observational study to investigate utility and issue of a platform that performs the online examination with monitoring using digital devices, online instruction on the use of drugs and delivering drugs for patients with Parkinson's disease (Care-4-One)  JAPIC-CTI Identifier: JapicCTI-205296^17^ | 2020 | | To assess patient satisfaction with a platform for online clinical care including home monitoring with digital devices, online patient compliance instruction, and prescription drug delivery for patients with PD | | Wearable activity tracker and recording device | Apple Watch Series 4, Apple Inc., iPhone 8 Plus, Apple Inc. | Kinematic parameters, wear-off, self-reported symptoms | 30 |
| Relationship between falls in Parkinson's disease patients and freezing feet in clinical and home situations  UMIN Identifier: UMIN000041373^44^ | 2020 | | To investigate the effect of FOG in rehabilitation and home settings on falls in patients with PD undergoing rehabilitation during their daily lives | | Wearable activity tracker | AX3 (Axivity Ltd) | Kinematic parameters | 50 |
| **Epilepsy** | |  | |  |  | | | |
| Seizure detection and warning system for epilepsy patients  ClinicalTrials.gov Identifier: NCT01874600^56^ | | 2013 | | To evaluate the effectiveness of a seizure detection and warning system | Wearable surface electromyographer | SPEAC System® (Brain Sentinel, Inc) | Electromyography data | 239^a^ |
| Research on development of wearable epilepsy monitoring device  UMIN Identifier: UMIN000030494^55^ | | 2017 | | To develop an epileptic seizure detection system | NR | NR | Heart rate, acceleration, respiration, electromyography, voice, body motion | 1000 |
| Promoting Implementation of Seizure Detection Devices in Epilepsy Care (PROMISE)  ClinicalTrials.gov Identifier: NCT03909984^54^ | | 2019 | | To test the performance of a wearable device for detection of seizures in patients with nocturnal epilepsy | Wearable heart rate and activity monitor | NightWatch (LivAssured B.V.) | Heart rate, activity | 60^a^ |
| Subcutaneous EEG: Forecasting of Epileptic Seizures (SUBER)  ClinicalTrials.gov Identifier: NCT04061707^53^ | 2019 | | To verify the efficacy of physiological indicators for predicting epileptic seizures using a wristwatch-type device | | NR | NR | Cardiac activity, heart rate, movement, muscle activity, skin potential, body temperature, and seizures | 10 |
| Non-invasive AI wearable device for supporting medical care  UMIN Identifier: UMIN000038690^45^ | | 2019 | | Prediction of episodic events related to symptoms of central nervous system disorders | NR | NR | NR | 100 |
| Utilizing Activity Trackers to Promote Physical Activity in People With Epilepsy: Can we Make a Difference?  ClinicalTrials.gov Identifier: NCT04357912^52^ | | 2020 | | To evaluate standard of care exercise education alone or in combination with a wearable physical activity tracker in people with epilepsy to determine the most effective way to increase physical activity and measure impact on depression, anxiety, quality of life, sleep, and seizure frequency | Wearable activity tracker  Fitness app | Fitbit  Stridekick app | Activity | 82 |
| Tele-epic (Telemedicine for Epilepsy Care) (Tele-epic)  ClinicalTrials.gov Identifier: NCT04496310^16^ | 2020 | | To facilitate management of epileptic seizures by collecting data on the level of antiepileptic drugs at home using a telemedicine device | | NR | NR | Serum drug levels | 600 |
| **Sleep disorders** | |  | |  |  |  |  |  |
| Sleep Health in Preschoolers: A Randomized Controlled Trial (SHIP)  ClinicalTrials.gov Identifier: NCT02255721^60^ | 2014 | | To verify the effect of the intervention by measuring the quality and quantity of sleep and aspects of sleep hygiene (pre-sleep activity/light exposure) using a wearable device in patients with sleep disorders in kindergarten children (30-71 months of age) | | NR | NR | Actigraphy  Light exposure | 500 |
| Evaluation of Spa Treatment on Insomnia (SOMNOTHERM)  ClinicalTrials.gov Identifier: NCT03991247^61^ | 2019 | | To test the effectiveness of an intervention by measuring sleep quality in patients with insomnia using a wristwatch | | Wearable wristwatch | NR | Actigraphy | 178 |
| The Impact of Insomnia on Pain in HIV (HIPPI)  ClinicalTrials.gov Identifier: NCT04298658^62^ | 2020 | | To investigate the impact of insomnia on pain, physical functioning, and inflammation in people living with HIV | | Wearable wristwatch | NR | Physical activity, sleep parameters, light exposure | 240 |
| Wake and Sleep State Transitions on a Portable Electroencephalogram (EEG) Device in Narcolepsy Type 1 (NT1) and Healthy Participants  ClinicalTrials.gov Identifier: NCT04445129^64^ | 2020 | | To determine whether portable devices can provide measurements at home similar to those taken in the clinical in participants with narcolepsy, and to investigate night-to-night changes in sleep patterns using these devices at home | | Portable EEG/ECG device or a wristwatch-type accelerometer | NR | EEG, ECG, activity | 45^a^ |
| Mind-Body Interventions to Mitigate Effects of Media Use on Sleep in Early Adolescents (Sleepazoid)  ClinicalTrials.gov Identifier: NCT04550507^63^ | 2020 | | To examine two related mind-body approaches -- mindfulness sensory awareness exercises and mindful body awareness check-ins -- in a randomized clinical trial of early adolescents with evening media use and sleep problems | | Wearable ECG and activity tracker | Empatica E4 (Empatica Inc), Actiheart (CamNtech Ltd) | ECG, activity | 75 |
| **Multiple sclerosis** | |  | |  |  |  |  |  |
| Dietary Approaches to Treat Multiple Sclerosis-Related Fatigue Study (Waves)  ClinicalTrials.gov Identifier: NCT02914964^73^ | 2016 | | To test the effectiveness of an intervention in patients with MS who are fatigued by measuring steps and sleep time with an accelerometer | | Wearable accelerometer | NR | Activity | 100 |
| Cognitive Behavioral Therapy, Modafinil, or Both for Multiple Sclerosis Fatigue (COMBO-MS)  ClinicalTrials.gov Identifier: NCT03621761^72^ | 2018 | | To compare the efficacy of 3 treatments for fatigue in MS: (i) telephone-based CBT, (ii) modafinil, and (iii) combination of CBT and modafinil | | Wearable electronic diary | PRO-Diary (CamNtech Ltd) | Self-reported fatigue intensity, activity level | 330 |
| The Impact of Training on Sitting Time and Brain Volumes in Multiple Sclerosis. (EXIMS)  ClinicalTrials.gov Identifier: NCT04191772^71^ | 2019 | | To explore the impact of a structured exercise program on sedentary time and health-related variables in patients with MS | | Wearable activity tracker | activPAL3 (PAL Technologies Ltd) | Activity | 156^a^ |
| Development of a Telehealth Obesity Intervention for Patients With MS: Modifying Diet and Exercise in MS (MoDEMS)  ClinicalTrials.gov Identifier: NCT04255953^70^ | 2020 | | To test the effectiveness of an intervention by measuring the amount of activity in patients with MS with obesity using an activity meter | | Wearable activity tracker | NR | Activity | 70 |
| Examining the Neural Effects of a Behavioral Intervention for Physical Activity in Multiple Sclerosis (BIPAMS-Brain)  ClinicalTrials.gov Identifier: NCT04496804^69^ | 2020 | | To assess the efficacy of a behavioral intervention that is based on social-cognitive theory and delivered through the Internet for increased physical activity | | Wearable activity tracker | NR | Activity | 24 |
| **Depression** | |  | |  |  |  |  |  |
| Lifestyle Intervention for Young Adults With Serious Mental Illness  ClinicalTrials.gov Identifier: NCT02815813^81^ | 2016 | | To evaluate the effectiveness of a group-based lifestyle intervention (PeerFIT) supported by mobile health (mHealth) technology and social media compared to Basic Education in fitness and nutrition supported by a wearable Activity Tracking device (BEAT) in achieving clinically significant improvements in weight loss and cardiorespiratory fitness in young adults with serious mental illness | | Wearable activity tracker | Fitbit (Fitbit Inc) | Activity | 150^a^ |
| Development of objective measures for depression, bipolar disorder and dementia by quantifying facial expression, body movement, and voice data during clinical interview and daily activity utilizing wearable device  UMIN Identifier: UMIN000023764^82^ | | 2016 | | To develop a machine learning algorithm that can objectively assess the severity of depression, manic-depressive illness, and dementia using facial expression, body movement, voice, and activity data | Wearable activity tracker | Silmee W20 (TDK Corp) | Activities of daily living | \| 500 \| No \| \| --- \| --- \| |
| Engaging Self-regulation Targets to Improve Mood and Weight and Understand Mechanism in Depressed and Obese Adults  ClinicalTrials.gov Identifier: NCT03841682^80^ | 2019 | | To test the effect of an intervention on obese patients with depression by measuring the number of steps with a wristwatch Fitbit | | Wearable activity tracker | Fitbit (Fitbit Inc) | Steps | 106^a^ |
| Preliminary study on just-in-time intervention using wearable devices aimed at improving sleep / waking rhythm against mood disorder  jRCT Identifier: jRCTs042190044^79^ | 2019 | | To verify the effect of the intervention by measuring sleep onset/wake time, sleep duration, and subjective health-related indices using wearable devices and apps for patients with mood disorders (depression, manic depression, dysthymia) | | NR | NR | Sleep parameters, subjective health-related indices | 30 |
| Internet-based behavioral activation therapy via smartphone for postnatal mothers to reduce postnatal depression and child abuse: a randomized controlled trial  UMIN Identifier: UMIN000036864^78^ | 2019 | | To evaluate the effects of the Japanese version of the Behavioral Acitvation Therapy Smartphone Program (Smart Mama) in postpartum women | | Smartphone | Smart Mama | Responses to tasks set by a therapist | \| 390 \| No \| \| --- \| --- \| |
| Smartwatch-supported Internet-delivered Intervention for Depression  ClinicalTrials.gov Identifier: NCT04568317^77^ | 2020 | | To investigate the acceptance of the use of a smartwatch in an internet-delivered CBT-based intervention for depression | | Smartwatch | NR | Activity, sleep parameters, self-reported mood | 70 |
| **Amyotrophic lateral sclerosis** | |  | |  |  |  |  |  |
| ALS Testing Through Home-based Outcome Measures (ALS AT HOME)  ClinicalTrials.gov Identifier: NCT03016897^85^ | 2017 | | To determine whether frequent home-based sampling can reduce the variability in measurements of activity, muscle, and fat | | Wearable activity tracker  Portable electrical impedance myography | NR  Skulpt® (Skulpt Inc.) | Actigraphy  Electrical impedance | \| 144^a^ \| No \| \| --- \| --- \| |
| Validation Of Tidal/End -Tidal CO2 in ALS (VOTECO2ALS)  ClinicalTrials.gov Identifier: NCT03764384^86^ | 2018 | | To evaluate the effectiveness of a hand-held device for detecting breathing failure in patients with motor neuron disease | | Portable respiratory monitoring platform | N-Tidal C (Cambridge Respiratory Innovations, Ltd) | End tidal CO_2_ (kPa), respiratory rate | 200 |

^a^ Actual sample size.

ALS, amyotrophic lateral sclerosis; CBT, cognitive behavioral therapy; ECG, electrocardiogram; EEG, electroencephalogram; FOG, freezing of gait; JAPIC-CTI, Japan Pharmaceutical Information Center Clinical Trials Information; jRCT, Japan Registry of Clinical Trials; MS, multiple sclerosis; NR, not reported; PD, Parkinson’s disease; UMIN, University Hospital Medical Information Network.
